# Development of Antioxidant COX-2 Inhibitors as Radioprotective Agents for Radiation Therapy—A Hypothesis-Driven Review

**DOI:** 10.3390/antiox5020014

**Published:** 2016-04-19

**Authors:** Markus Laube, Torsten Kniess, Jens Pietzsch

**Affiliations:** 1 Department of Radiopharmaceutical and Chemical Biology, Institute of Radiopharmaceutical Cancer Research, Helmholtz-Zentrum Dresden-Rossendorf, Bautzner Landstrasse 400, Dresden D-01328, Germany; t.kniess@hzdr.de; 2 Department of Chemistry and Food Chemistry, Technische Universität Dresden, Dresden D-01062, Germany

**Keywords:** COX-2 inhibitors (COXIBs), cyclooxygenases, normal tissue, nonsteroidal anti-inflammatory drugs (NSAIDS), oxidative stress, radiation-induced vascular dysfunction, radioprotection, radiosensitization, reactive oxygen/nitrogen species, tumor models

## Abstract

Radiation therapy (RT) evolved to be a primary treatment modality for cancer patients. Unfortunately, the cure or relief of symptoms is still accompanied by radiation-induced side effects with severe acute and late pathophysiological consequences. Inhibitors of cyclooxygenase-2 (COX-2) are potentially useful in this regard because radioprotection of normal tissue and/or radiosensitizing effects on tumor tissue have been described for several compounds of this structurally diverse class. This review aims to substantiate the hypothesis that antioxidant COX-2 inhibitors are promising radioprotectants because of intercepting radiation-induced oxidative stress and inflammation in normal tissue, especially the vascular system. For this, literature reporting on COX inhibitors exerting radioprotective and/or radiosensitizing action as well as on antioxidant COX inhibitors will be reviewed comprehensively with the aim to find cross-points of both and, by that, stimulate further research in the field of radioprotective agents.

## 1. Introductory Remarks

As recently emphasized by Adam and Kenny, radiation therapy (RT) evolved to be a highly sophisticated, cost-effective, primary method of treatment for patients with cancer, curing many of them or being very effective in the relief of symptoms if curative treatment is not feasible [1]. Currently, more than 50% of cancer patients receive RT, often used in combination with surgery and chemotherapy. However, clinical RT using external beam treatment with photons or particles, brachytherapy and/or radionuclides strongly demands consideration of radiation damage to healthy tissue [2]. A continuous effort is going on by researchers and clinicians to improve dose delivery to cancer tissue while limiting adverse reactions in the adjacent normal tissues, and by that to broaden the therapeutic window. On the one hand, this comprises the use of fractionated/intensity modulated radiotherapy schemes or novel treatment techniques minimizing the volumes of irradiated normal tissues [1,3]. On the other hand, this involves the development of potent radioprotective agents, a topic which just recently has been comprehensively reviewed in the literature [4,5,6,7]. However, despite a variety of promising compounds from several substance classes has been evaluated and broad data bases on their pharmacological and radiobiological properties are available so far only two radioprotective compounds, amifostine and palifermine, have found their way into the clinics [8]. As an own contribution to debate this topic scientifically we hypothesize and gather evidence on antioxidant COX-2 inhibitors to be promising radioprotectants.

## 2. Biochemical Background

RT and, thereby, the exposure of tissue to ionizing radiation (IR), causes damage of DNA, proteins, and lipid membranes leading to cell dysfunction or even cell death [9]. This is mediated by direct effects, like DNA single or double strand breaks, and indirect effects, as the destruction of biomolecules caused by reactive oxygen as well as reactive nitrogen species (ROS and RNS, respectively) resulting from the radiolysis of water. Hydroxyl radicals (^•^OH), hydrogen peroxide (H_2_O_2_), superoxide radical anions (O_2_^•−^), and peroxyl radicals (ROO^•^) as well as nitric oxide (NO^•^) and peroxynitrite (ONOO^−^) are only some of the relevant ROS and RNS products. The impact of these immediate radiolysis products is considerable, e.g., 60%–70% of cellular DNA damage is caused by the highly reactive and thus locally acting ^•^OH [10]. The endogenous cellular redox system, enzymatic (superoxide dismutase (SOD), glutathione peroxidase (GSH-Px), catalase, and peroxiredoxin) as well as non-enzymatic (vitamin C, uric acid, and glutathione (GSH)), is capable to maintain redox homeostasis to a certain extent [10]. However, therapeutic doses of IR exceed this threshold to provoke the intended radiation toxicity in tumor tissue but coincidently induce several unwanted acute and chronic side effects in normal tissue. Besides components of the “redox system”, active secretion or “apoptotic” release of cytokines, chemokines, and damage (or danger)-associated molecular pattern molecules (DAMPs) by irradiated cells, stimulates local and complex systemic responses in non-irradiated cells. These mediators are involved in triggering systemic stress, e.g., by activation of nuclear factor κB (NF-κB)-mediated pathways, via cytokine and/or toll-like receptors [11,12,13]. Starting from the 1960s, studies uncovered that radiation exposure might increase the risk for other complications, including stroke as well as heart, digestive, and respiratory diseases [14]. Exemplarily, there is a body of evidence that irradiation during tumor therapy effects the vascular system as well, leading to inflammatory reactions during the acute treatment and accelerated atherosclerosis in the late phase. For example, irradiation treated head and neck cancer or breast cancer patients are at higher risk to suffer from cerebrovascular and cardiovascular complications after >5–10 years [14,15]. This to some extent limits RT of cancer by shifting the risk of dying from cancer to an increased risk of cardiovascular diseases. As one consequence, in modern irradiation protocols the irradiated volume of the heart and major coronary vessels is reduced, but a distinct radiation exposure is unavoidable. In this regard, strategies to protect patients from these late effects are highly desirable [16]. The response of normal tissues comprises two main components. The first resembles the healing process of traumatic wounds, *i.e.*, activation of the coagulation system, inflammation, epithelial regeneration, granulation tissue formation, and matrix deposition and remodeling. The second resembles specific injuries that are responsible for the progression of injury over many years (both reviewed in [17]). For both components, a complex network of inflammatory and immune processes plays a key role [12,15,18,19,20,21].

Within this network, here selected exemplary, as a consequence of ROS-mediated activation of a cascade of enzymes (e.g., PLA_2_) and transcription factors (e.g., NF-κB; E2 promoter-binding factor-1, E2F-1; hypoxia-inducible factor 2α, HIF-2α) [22,23], the radiation exposure causes the excessive production of COX-mediated eicosanoids such as prostaglandins and thromboxane but also 5-lipoxygenase (5-LOX) mediated leukotrienes. The increased levels of prostaglandins and thromboxane, and thus an inflammatory state in the tissue, can arise within hours and persist up to weeks after irradiation in a wide range of organs and tissues as shown in experimental studies [24]. The vascular endothelium is also affected by ionizing radiation what is recognized as one important cause of radiation induced late effects. The kind of injury depends on vessel structure. Microthrombi and tissue ischemia are caused by an inflammatory reaction in the microcirculation. In larger arteries -in the presence of high cholesterol- monocyte invasion and transformation into activated macrophages can occur thereby initiating atherosclerosis [15]. Protection of endothelial cells against ROS is to a certain extent achieved by endogenous antioxidants like GSH and SOD. However, endothelial cells appear to be the most radiosensitive elements in the vessel wall, maybe even among the fixed cells of the mesenchyme [25]. Thus, protection of the endothelium is of importance to ameliorate radiation induced vascular late effects. Interventions to protect normal tissue can in principle be divided in three groups based on the time scale of events taking place after exposure to ionizing radiation [26]. First, chemical radiation protectors intervene in the immediate events after energy absorption (10^−17^ to 10^−13^ s after IR impact) comprising the excitation and ionization of atoms and the formation of ^•^OH near (target) molecules (10^−10^ to 10^−6^ s), and the formation of DNA radicals (10^−6^ to 10^−3^ s). Radioprotective chemical drugs have been intensively investigated in the past with the aim to protect workers and soldiers from radiation toxic effects of nuclear accidents or patients from normal tissue side effects in the course of RT. These efforts have generated a library of different compounds comprising synthetic thiols and phosphothioates, naturally occurring antioxidants, and antioxidant drugs [27]. One of the most effective compounds is amifostine (WR-2721, *S*-2(3-aminopropylamino)ethyl phosphorothioic acid) having a dose reduction factor of 2.7 in a mouse 30-day lethality model and being a U.S. Food and Drug Administration (FDA) approved radioprotector (used to ameliorate xerostomia during RT), today [9]. Recently, palifermin, a truncated derivative of keratinocyte growth factor, has been approved by the FDA and is used in clinical trials as a radioprotective agent [28]. In comparison, for naturally occurring antioxidants like GSH, SOD, melatonin, or antioxidant vitamins A, C, and E, dose reduction factors of 1.3 or lower are reported and their effectivity is generally limited to lower doses or dose rates of radiation exposure [27]. Second, radiation mitigators have been investigated, compounds that modulate the enzymatic repair of oxidized DNA and DNA strand breaks (seconds to hours after IR exposure) as well as influence cell proliferation or degeneration followed by cell death or cell survival under mutation of cells and carcinogenesis (hours to years after IR exposure). The third group comprises the (usual) treatment of late effects like fibrosis, scarring, vascular damage, and organ damage (weeks to years after IR exposure). As seen from the time scales, the second and third interventions cannot be kept apart in every aspect, thus also comprising similar intervention approaches, e.g., the modulation of signal transduction, gene expression, host cell activation, inflammation, repopulation, and proliferation. In this regard, compounds from several drug classes have been identified as potential radiation mitigators, e.g., growth factors, protease inhibitors, angiotensin-converting-enzyme (ACE) inhibitors, isoflavones, 3-hydroxy-3-methylglutaryl-CoA (HMG-CoA) reductase inhibitors, and COX-2 inhibitors and NSAIDs [26,29].

Unfortunately, none of these agents has been suitable to prevent all damage of normal tissue and to protect the endothelium completely so it was pointed out by Fajardo *et al.* that the vascular injury is a price to be paid for the success of cancer radiotherapy [25]. However, with respect to the clinical situation the development and evaluation of novel drug candidates or new strategies is of urgent need. Our own investigations provided experimental evidence on 2,3-diaryl-substituted indole-based COX-2 inhibitors exerting at pharmacologically low concentration levels antioxidant activity, for instance by scavenging ^•^OH and O_2_^•−^ thus protecting low density lipoproteins from oxidative damage as well as demonstrating radioprotection on both cellular and organotypical vascular models [30,31]. From these data we deduced the hypothesis that antioxidant COX-2 inhibitors can be considered to act as a double-edged sword by intervening in the immediate and also delayed responses to ionizing radiation. Compounds of this class able to protect especially lipid membranes, e.g., in endothelial cells, are reckoned compounds to reduce radiation induced vascular late effects.

## 3. Cyclooxygenase-2 and COX-2 Inhibitors

Cyclooxygenase-2 (COX-2; EC 1.14.99.1) is the isoform of cyclooxygenases which is mainly responsible for the time-dependent and localized production of prostaglandins at inflammatory sites, *i.e.*, also after tissue exposure to ionizing radiation. COX-2 overexpression has been implicated in a number of diseases such as chronic inflammatory diseases like rheumatoid arthritis, neurodegenerative disorders like Parkinson, and a variety of cancer entities [32,33,34]. Several unique properties emphasize this enzyme as an interesting and drug-able target for therapeutic approaches [35]. Cyclooxygenase-2 (i) catalyzes the key step in the formation of prostanoids and thus provides the autocrine and paracrine acting mediators to regulate the inflammatory response; (ii) is nearly absent in most tissues under physiological conditions but its expression is induced locally by inflammatory and proliferative stimuli and (iii) its overexpression has been associated with radioresistance of tumors as well as radiation-induced inflammation implying in both beneficial results of COX inhibition [36,37,38,39]. Besides triggering the inflammatory response itself, COX-2 activity has also been suggested to decrease cells reducing power by depletion of GSH (needed to reduce prostaglandin G_2_ (PGG_2_) to prostaglandin H_2_ (PGH_2_)) [40] as well as to increase Fe^2+^-toxicity in neuronal cells via generation of O_2_^•−^ [41]. COX-2 exists beside COX-1, the constitutively expressed isoform of cyclooxygenases which produces prostanoids mainly to maintain endogenous homeostasis. Both enzymes catalyze the conversion of arachidonic acid (AA) to PGH_2_ by the same two step reaction, *i.e.*, the oxidation of AA in the cyclooxygenase-active site forming PGG_2_ and the reduction of this intermediary peroxide to the respective alcohol PGH_2_ at the peroxidase-active site [42,43]. The cyclooxygenase-active site is addressed for the inhibition of both COX isoforms. However, isoform-selectivity in favor of COX-2 inhibition, as an important determinant for this class of compounds, can be achieved by taking advantage of the larger volume of cyclooxygenase-active site of COX-2 and an additional accessible side pocket therein. Examples for clinically approved inhibitors represent the nonsteroidal anti-inflammatory drugs (NSAIDs) like aspirin or ibuprofen and the selective COX-2 inhibitors (COXIBs), e.g., celecoxib. In terms of chemical structure, NSAIDs comprise among many others salicylates, arylacetic acids, and phenylpropionic acids. Most COXIBs can be assigned to the class of diaryl-substituted heterocycles bearing a methylsulfonyl or aminosulfonyl group at one of the phenyl rings [44,45,46].

Beside antioxidant properties of COX inhibitors, prooxidant activity of COXIBs has been discussed. The increase of susceptibility of human low density lipoprotein (LDL) to oxidative modification by the methylsulfonyl-substituted COXIBs rofecoxib and etoricoxib at pharmacologically relevant concentrations has been related to the interaction of the inhibitors with the membrane phospholipids using small-angle X-ray diffraction by Walter *et al.* [47]. The authors hypothesized that the location of rofecoxib near the head group region of the phospholipid bilayer results in increased permeability for free radical ion and free radical diffusion and thus susceptibility to oxidation. In contrast, the sulfonamide-substituted COXIBs celecoxib and valdecoxib did not show such a prooxidant effect in this setting which was consistent with their location in the upper region of the hydrocarbon chains adjacent to the phospholipid headgroups [47]. Furthermore, rofecoxib can undergo an unique oxidation chemistry with regard to other COXIBs [48]. The central furan-2(5*H*)-one core can be deprotonated in aqueous solution forming a reactive rofecoxib anion which is prone to oxidation by molecular oxygen forming a maleic anhydride. The respective intermediary radical species is believed to promote lipid oxidation in low density lipoproteins [49]. Both prooxidant actions and a class effect of COX-2 inhibitors on thromboxane A_2_/prostaglandin I_2_ levels in the vasculature are discussed to cause cardiovascular side effects after long-term treatment, reasoning the withdrawal of rofecoxib and valdecoxib from the market [34,50,51]. Noteworthy, arachidonic acid also forms by non-enzymatic free-radical catalyzed peroxidation stable prostaglandin-like products, the isoprostanes. Among them, F_2_-isoprostanes have been identified as reliable markers for oxidative stress *in vivo*, e.g., in inflammatory and atherogenic processes, and were discussed as pathophysiological mediators of oxidant injury [52,53,54,55].

## 4. COX-2 Inhibitors as Radiosensitizers and Radioprotectors

**Figure 1:**
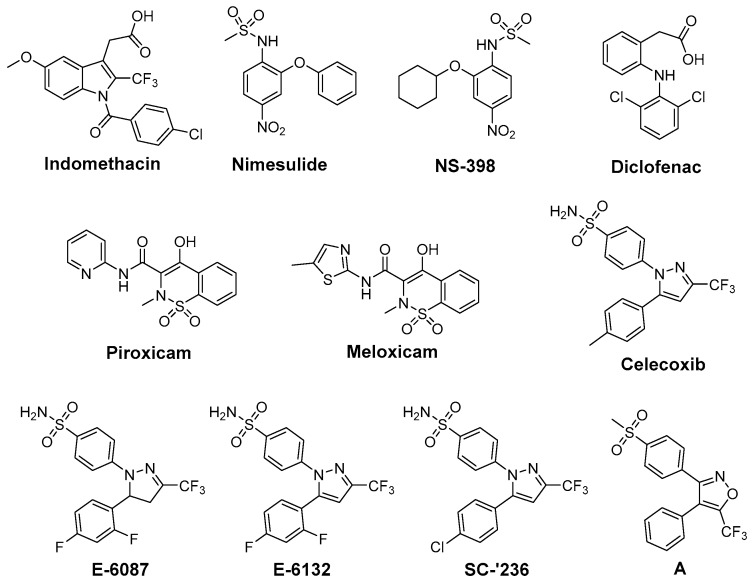
COX-2 inhibitors showing radiosensitizing effects in experimental tumor models.

One approach to protect normal tissue and to achieve a better tumor response to RT, respectively, is to spare the needed radiation dose by applying radiosensitizing drugs (Figure 1). Therefore, COX-2 inhibitors have been evaluated regarding their radiosensitizing activity because several studies showed that COX-2 expression of the tumor is closely correlated with radioresistance and hence the outcome of RT [56]. First studies in this field showed indomethacin enhancing the tumor growth delay and curability in RT treated mice bearing immunogenic methylcholanthrene-induced fibrosarcoma and non-immunogenic spontaneous fibrosarcoma tumors [57]. Further *in vivo* studies using this [58,59,60,61] and other tumor models as glioblastoma [62,63,64,65], secondary bone tumors [66], and lung A549 tumor xenografts [67] unraveled in the following years radiosensitization also for other COX-2 selective inhibitors such as nimesulide [67], NS-398 [59], celecoxib [58,63,64,66], and the celecoxib derivatives E-6087, E-6132 [65], SC-′236 [60,61,62] (reviewed in part in [39,56,68]). Inhibition of intratumoral PGE_2_ synthesis by celecoxib [69], an increase of tumor oxygenation by piroxicam, indomethacin, diclofenac, and NS-398 [59], and anti-angiogenic action of celecoxib [63,66] were related to the increase in radiosensitivity *in vivo*.

Furthermore, several *in vitro* studies were performed to elucidate the underlying mechanism of radiosensitization in different cell lines [67,70,71,72,73,74,75,76,77,78,79,80]. Beside the above mentioned inhibitors, also meloxicam [81] and the valdecoxib derivative **A** [82] were found to act as radiosensitizers in these studies. Inhibition of PGE_2_ synthesis, reduction of COX-2 expression in HeLa cells by celecoxib [70], inhibition of DNA repair in HN5 cells by celecoxib [71], and arrest of cells in the radiosensitive G2M phase [80] have been described as COX dependent mechanisms. However, also COX-2 independent mechanisms like inhibition of nuclear endothelial growth factor receptor accumulation in A459, HCT116, and HSF7 cells [73] or reduction of vascular endothelial growth factor C protein expression in HeLa cells by celecoxib [70] are underlying the radiosensitizing effect of COX-2 inhibitors. Further examples are the downregulation of β-catenin in highly radioresistant Eca109R50Gy cells by NS-398 [72] and the radiation-induced apoptosis by caspase-mediated apoptotic signals in A549 cells triggered by nimesulide [67]. It should be noted that also pairings of COX-2 inhibitors and non-responding cell-lines have been described, namely celecoxib and human prostate cancer cell lines PC-3, DU145, and LNCaP [74], NS-398 and prostate carcinoma PC-3 cells [75], and nimesulide and head-and-neck carcinoma cells SSC9 and SCC25 [76]. These findings might be related to the fact that the dominant mechanism for radiosensitization is likely tumor cell line dependent [77]. However, concentration related effects might also play a role as described for celecoxib in HeLa cells [70] and prostate cancer cell lines PC-3, DU145, and LNCaP [78]. The combination of COX-2 inhibition with chemotherapy and RT was also investigated revealing further increases in radiosensitivity, e.g., by application of celecoxib, docetaxel, and irradiation in A431 human tumor xenografts in mice [77]. Clinical trials combining COX-2 inhibition and RT [83] or COX-2 inhibition, chemotherapy (5-fluorouracil [84,85], tegafur-uracil and folinate [86], or erlotinib [87]), and RT have also been performed which revealed safety of the treatment combined with improved outcome in some but also not significantly improved outcome in most cases (reviewed in [88]).

**Figure 2:**
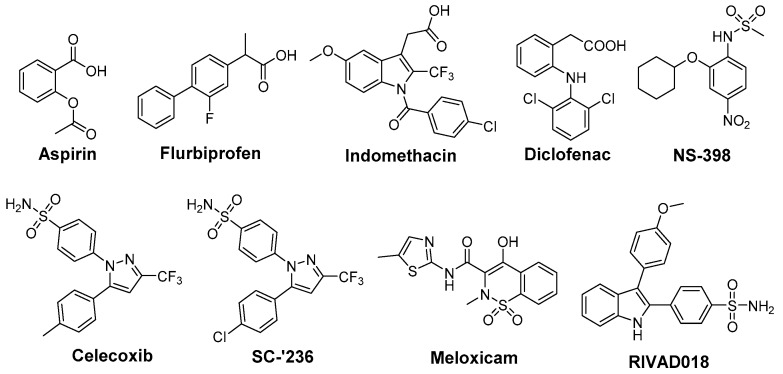
COX-2 inhibitors with radioprotective effects.

In opposite, radioprotective agents target normal tissue and minimize radiation-induced toxicity (Figure 2). A variety of COX inhibitors has been investigated regarding their efficacy in this mind (reviewed under different aspects in [24,89,90,91,92]). Aspirin was found to reduce radiation-induced renal functional damage in mice after two irradiations but with clinically relevant fractionated irradiation schedules no significant reduction could be observed [93,94]. Recently, aspirin was evaluated in whole-body irradiated rats revealing antioxidant effects on malondialdehyde (MDA) levels as well as myeloperoxidase levels but no differences in histopathology were observed in lung tissue after single whole-body irradiation with a median lethal dose [95]. The clinical evidence for radioprotective action for aspirin is not conclusive. Effectivity is indicated by studies showing the amelioration of RT-induced diarrhea in the course of gynecological malignancy therapy in 28 patients treated either with high dose aspirin or placebo [96] and in treatment of epithelial ulceration resulting from pelvic irradiation in 10 patients [97]. However, a comprehensive trial investigating the effect of aspirin on acute and late effects after conservative surgery for early breast cancer and irradiation did not reveal any effect in 186 women treated with either aspirin or placebo starting 1 day before and lasting for 1 year following radiotherapy [98]. Cu(II)_2_ (3,5-diisopropylsalicylate)_4_, sharing the salicylic acid motif with aspirin, was found to increase survival in whole-body irradiated mice and to exert SOD mimicking activity [99,100].

Indomethacin, an indole-based NSAID, was evaluated regarding its radioprotective effects in several tissues [24]. Preclinical studies revealed protection against radiation-induced damage of the alimentary tract in mice [101,102], rat [103], dog [104], and opossum [105] but also a lack of protection in a number of settings [57,106]. Furthermore, radioprotection of hematopoietic tissue in mice [57], parotic glands [107] and of the central nervous system [108,109] in rats, and of the skin in guinea pigs [110] was observed. The effect of indomethacin on protection for irradiation esophagitis during lung cancer treatment by irradiation with a total dose of 40–60 Gy was investigated in a double-blind study with 28 patients receiving either indomethacin or placebo. Although histopathological findings did not differ, the symptomatology with indomethacin was milder and fewer patients suffered from esophagitis scored 2 or higher by endoscopy [111]. Indomethacin also showed protection against severe oral mucositis but did not increase the overall 2 year survival in a double-blinded study with 19 head and neck cancer patients treated with RT and indomethacin or placebo [112]. Flurbiprofen also showed radioprotection in preclinical, for example in rabbit eye [113], and in clinical studies, e.g., delaying the onset of mucositis and reducing the severity at two weeks after RT in 12 head and neck cancer patients, but the overall severity or duration of mucositis was not improved [114]. Several COX inhibitors were evaluated regarding their stimulating action on hematopoiesis recovery after whole-body X-ray irradiation because of the important role of PGE_2_ synthesis inhibition in the negative hematopoietic feedback control. Non-selective COX-2 inhibitors like indomethacin, diclofenac, and flurbiprofen were evaluated in different experimental settings showing stimulatory action on hematopoiesis after irradiation [89,115]. However, the increase in the survival rate after bone marrow irradiation could not be translated to experiments of bone marrow and gastrointestinal tract irradiation due to the gastrointestinal toxicity of the non-selective COX inhibitors indomethacin and diclofenac [89,116,117]. One approach to counteract gastrointestinal toxicity of NSAIDs and the cardiovascular side effect of COXIBs is the development of respective NO-releasing drugs. In this context, the nitroxybutyl ester of flurbiprofen was evaluated in sublethally and lethally irradiated mice but no further improvement neither on hematopoietic parameters nor the overall survival rate compared to the parent drug flurbiprofen was observed [118]. COX-2 selective inhibitors lack the gastrointestinal toxicity of classical NSAIDs by principle, *i.e.*, not inhibiting COX-1 which is also responsible for homeostasis in the gastric mucosa. Therefore, meloxicam has been extensively investigated in the recent years [89]. For instance, meloxicam, administered either before or repeatedly after irradiation exposure, has enhanced the recovery of hematopoietic progenitor cells committed to granulocyte-macrophage and erythroid development in sublethally irradiated mice [119]. Interestingly, a single dose of meloxicam increased the survival rate only if applied before but not after lethal whole-body irradiation suggesting, as discussed by the authors, either vascular or hepatic side effects by repeated treatment with meloxicam or the dependency on an induction of granulocyte colony-stimulating factor (G-CSF) production promoting cell survival by suppressing apoptosis [120]. The combination with additional treatment strategies, namely administration of meloxicam and G-CSF or IB-MECA, an adenosine A_3_ receptor agonist, increased the effect on hematopoiesis and survival rate in comparison to the treatment with each single drug [121]. A similar effect was observed for other NSAIDs as well; e.g., for indomethacin in combination with muramyl tripeptidephosphatidyl ethanolamine [122] or broncho-vaxom [123]. Meloxicam was investigated also in a glioblastoma multiforme tumor model because rapid recurrence of this malignancy after resection of the primary tumor has been related to the radiation induced stimulation of infiltration of glioma cells into healthy brain tissue and attributed partially to neuro-inflammatory processes. In either non-irradiated or irradiated rat brains, the effect of meloxicam treatment on stimulation of F98 glioma tumor growth was determined. Meloxicam was shown to largely prevent irradiation induced PGE_2_ production and matrix metalloproteinase-2 activity, and in turn to prevent the stimulation of F98 cell infiltration leading to a median survival time comparable with non-irradiated rats [124]. In a number of studies celecoxib was investigated as radioprotector. Radiation pneumonitis is a severe and dose limiting side effect in lung cancer treatment. In this regard, celecoxib was tested in rats after single dose X-ray irradiation of the right hemithorax and mediastinal region with 20 Gy revealing a dose dependent protective effect on lipid peroxidation (MDA levels) and histopathological parameters [125]. Celecoxib and diclofenac were compared in a rat model combining a dose of 2 Gy under acute inflammation [126] and adjuvant-induced arthritis [127], respectively. For both inhibitors significant inhibition of the inflammatory response was found, *i.e.*, volume reduction of inflamed paw, decrease of PGE_2_ and other inflammatory cytokines like IL-6, IL-1β, and TNF-α, but no effect on radiation induced lipid peroxidation could be determined. A decrease in severe skin reactions after irradiation of cutaneous tissue at a high single dose of 50 Gy in celecoxib pre- or post-treated mice was also observed [128]. Oral mucositis is another inflammatory, severe, and dose limiting side effect of RT, namely of advanced head and neck cancer, and Haagen *et al.* evaluated celecoxib’s radioprotective effects in a mouse tongue model [129]. Although COX-2 was found to be implied in the epithelial response to irradiation, celecoxib did not affect the epithelial ulcerative response in any of the tested protocols comprising single or fractioned irradiation. This suggests that inflammatory pathways were not relevant for changes of epithelial radiation tolerance in this setting [129]. A similar result was observed in a double-blind placebo-controlled clinical trial including 40 head and neck cancer patients treated with celecoxib or placebo during the course of RT revealing no reduction in the severity of oral mucositis and pain [130]. In mice, celecoxib and meloxicam were tested in a setting of combined injury, an insult of radiation injury and coincident tissue trauma. Both inhibitors were shown to have no therapeutic value and meloxicam even increased mortality of wounded and combined injury mice. The authors concluded that in mice treatment of combined injury with these COX-2 inhibitors is contraindicated [131].

NS-398 was found to decrease radiation-enhanced breast cancer cell invasion of MDA-MB-231 cells induced by irradiated 3T3 fibroblasts suggesting diminished invasiveness of surviving breast cancer cells after RT [132]. NS-398 was also found to reduce the bystander effect in human lung fibroblasts [133]. Interestingly, NS-398 induced tumor cell arrest at G1 phase of the cell cycle and inhibited expression of transcription factors pRb and E2F-1 and, subsequently, of COX-2 in squamous cell carcinoma cells [134]. The celecoxib derivative SC-′236 only slightly increased the response of jejunal mucosa and had no effect on severity of fibrosis causing leg contracture after whole-body irradiation [60]. Another COX-2 selective indomethacin amide derivative only showed a slight effect on radiation induced formation of isoprostanes at a 10 Gy dose in human aortic endothelial cells *in vitro* [135].

## 5. 2,3-Diaryl-Substituted Indoles as Promising Radioprotectors

**Figure 3:**
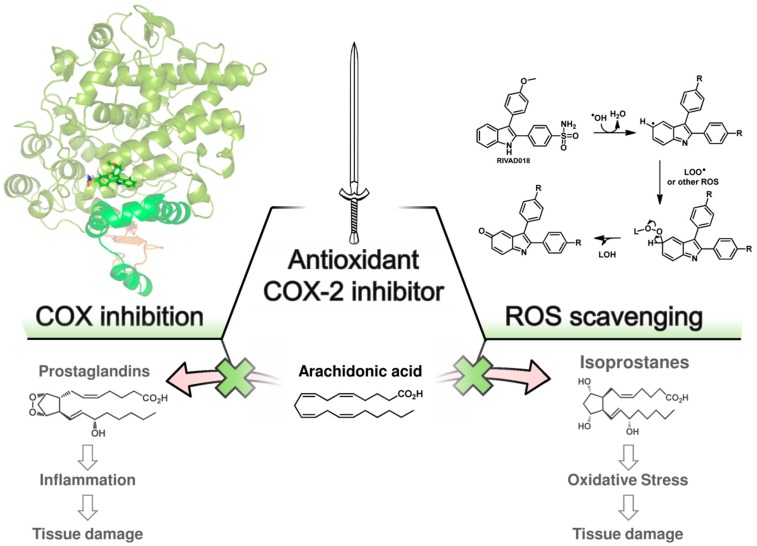
Proposed double-edged sword concept of action of antioxidant COX-2 inhibitors. Antioxidant COX-2 inhibitors exert radioprotection by inhibition of COX-2 activity and ROS scavenging activity. COX-2 inhibitors block prostaglandin synthesis by binding in the cyclooxygenase active site of COX-2. The suggested binding mode of **RIVAD018** in the COX-2 enzyme is shown (**left**, crystal structure protein data base (PDB) entry 3ln1, modeling as recently reported by us [149]); ROS scavenging of 2,3-diphenyl-based COXIBs is suggested to follow a mechanism initialized by hydrogen abstraction at C-5 of the indole by ^•^OH forming a carbon-centered radical followed by the reaction with other ROS, e.g., O_2_^•−^, LOO^•^, or NO^•^, and formation of indol-5-one derivatives after rearrangement processes (**right**, mechanism according to [143,144]).

We have performed radioprotection studies with the sulfonyl-containing 2,3-diaryl-substituted indole **RIVAD018** and observed an effect both in cellular and organotypical vascular models [30,31]. The 2,3-diaryl-substituted indoles have been initially developed by Hu and Guo *et al.* as potent COX-2 selective inhibitors [136,137,138]. Based on that, our group reported on the radiosynthesis of a fluorine-18 labelled derivative [139] and the *in vitro* and *in vivo* investigations on an autofluorescent potent candidate of this class [140,141] with the aim to get access to COX-2 targeted imaging agents for *in vitro* and *in vivo* applications. Taking advantage of varying the substitution pattern at the C-5 position as well as the phenyl rings at C-2 and C-3 of the indole, we developed a set of novel derivatives and identified within this class potent antioxidant COX-2 inhibitors that protected proteins and lipids in low density lipoproteins from oxidation with a potency comparable to or better than the well-known antioxidant melatonin [142]. Compound **RIVAD018** bearing an aminosulfonyl-substituted and a methoxy-substituted phenyl ring was found to be the most potent antioxidant prolonging the lag phase of copper-induced lipid peroxidation by factor 2.85 in comparison to 1.71 for melatonin at 1 µM concentration. A slightly weaker antioxidative capacity of **RIVAD018** was observed in the iron-catalyzed protein oxidation of LDL, *i.e.*, 1.98-fold prolongation of lag time in comparison to 1.52-fold by melatonin. Furthermore, a more detailed investigation on scavenging of ^•^OH, O_2_^•−^, and hypochlorite (OCl^−^) by selected derivatives of the 2,3-diaryl-substituted indoles revealed a preferred interaction with transition-metal catalyzed oxidation compared to HOCl-mediated oxidation, and preferred ^•^OH compared to O_2_^•−^ scavenging [31]. The mechanism of antioxidant capacity is suggested to follow a mechanism shown in Figure 3 introduced by Antosiewicz *et al.* [143] for structurally similar nitroxides and suggested by Suzen *et al.* [144] for 2-phenyl indoles as well. To illustrate the biological relevance of this finding, we have examined oxidatively modified LDL and their 2,3-diphenyl-1*H*-indole protected counterparts *in vitro* regarding scavenger receptor mediated uptake in cells, oxidized lipoprotein mediated cell adhesion, and oxidized lipoprotein-mediated inflammatory response as well as *in vivo* regarding the influence on the biodistribution of the LDL particles. Consistently throughout these experiments, 2,3-diarylindole protected LDL showed characteristics similar to unmodified LDL verifying the substantial protection against ROS induced damage of LDL [31].

Based on the importance of ROS for radiation induced acute and late effects, we tested **RIVAD018** regarding its radioprotective effects. Antioxidant 2,3-diarylindole **RIVAD018** was initially evaluated in terms radioprotection using human arterial (HAEC) and microvascular (HDMEC) endothelial cells exposed to moderate doses (sham, 2 and 10 Gy) of X-ray radiation in the presence or absence of **RIVAD018** as well as other redox inactive COX-2 inhibitors, respectively. In contrast to IR-induced COX-2 expression and activity (measured by PGE_2_ release) which was markedly decreased by all COX-2 inhibitors, **RIVAD018** alone was able to block the formation of the vasoactive isoprostanes 8-iso-PGE_2_ and 8-iso-PGF_2α_, and to reduce cytokine levels substantially. The anti-inflammatory and antioxidant intervention in these autocrine and paracrine activation loops of the vascular endothelial cells suggest that antioxidant COX-2 inhibitors may act as radioprotectors to reduce radiation-induced vascular dysfunction [135,145]. With the aim to evaluate the radioprotective effects of 2,3-diarylindoles in an organotypical vascular system, **RIVAD018** was furthermore evaluated in a rat aortic ring model [30,146]. For that, rat aortas obtained from male Wistar Unilever rats were dissected in small rings and irradiated using moderate doses (sham, 4 and 10 Gy) of X-ray radiation in the presence of 1 µM **RIVAD018** and celecoxib, respectively. The acute and subacute effects were determined at day 1 respectively 3 post irradiation (i) on COX expression level; (ii) on functional expression of COX-2 by measuring uptake of an ^11^C-labeled COX-2 selective inhibitor [147]; and (iii) on COX-dependent formation of PGE_2_ and COX-independent, ROS-derived formation of 8-iso-15(R)-PGF_2α_. Consistent with an induction of COX-2 by IR, a dose dependent increase of COX-2 but not COX-1 expression was observed. COX-2 expression was localized in the endothelium and, additionally at 10 Gy, in the smooth muscle cells building up the tunica media of the rat aortic rings. PGE_2_ and 8-iso-15(R)-PGF_2α_ levels in the supernatants of the aortic rings increased in a dose dependent manner. Celecoxib was only effective to inhibit PGE_2_ formation but not to decrease 8-iso-15(R)-PGF_2α_ levels. In contrast to celecoxib, **RIVAD018** was able to block PGE_2_ as well as 8-iso-15(R)-PGF_2α_ formation [30,146]. In this context, F_2α_-isoprostanes deriving from non-enzymatic oxidation processes were shown to act as reliable markers of *in vivo* oxidative stress [55,148]. Thus, substantial reduction of isoprostanes by **RIVAD018** under the experimental conditions employed also can be clearly attributed to the high antioxidative potential of 2,3-diaryl-substituted indoles. In summary, **RIVAD018** exerts radioprotective effects based on COX-2 inhibition and ROS scavenging also in this complex organotypical model.

Based on these experiments we hypothesize that antioxidant COX-2 inhibitors show bifunctional action on two essential pathways of radiation induced normal tissue injury, acting metaphorically speaking as a double-edged sword. Up to now, the effect of COX inhibitors on antioxidant parameters during and after radiotherapy has been described only in two cases (aspirin [95], celecoxib [125]) and, as one conclusion of the authors, a wide variety of antioxidant COX inhibitors known from the literature have to be thoroughly investigated in the future in this regard.

## 6. Antioxidant COX-2 Inhibitors as an Extensive Library for Promising Radioprotectants

Several COX inhibitors have been evaluated regarding their radioprotective effects so far but the application as antioxidant in preclinical and clinical RT settings seems rare. Since a wide number of COX-inhibitors were found to exert antioxidant activity, which are in principle available to be used as promising radioprotectors, we will give in this section an overview about these chemically interesting compounds.

**Figure 4:**
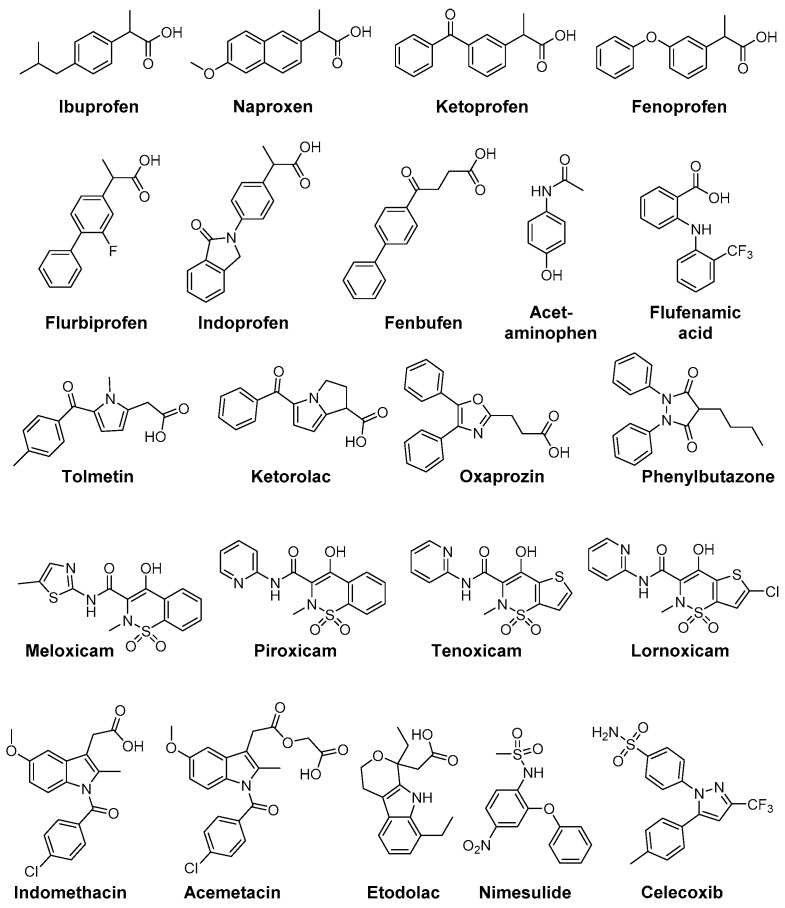
Clinically used NSAIDs and COXIBs with antioxidant capacity.

Increased levels of reactive oxygen species respectively signs of oxidative stress (protein oxidation and lipid peroxidation) have been found in a number of pathologies like rheumatoid arthritis, diabetes, cardiovascular diseases as atherosclerosis or hypertension, and neurodegenerative disorders like Parkinson’s or Alzheimer’s disease [150]. For example, osteoarthritis (OA) is a multifactorial disease and the underlying pathophysiological processes comprise the release of AA by PLA_2_ from damaged cells and subsequent conversion to inflammatory mediators by COX and 5-LOX enzymes as well as higher levels of oxidative species and antioxidant enzymes in the synovial fluids [151]. Due to the importance of ROS for these in part also inflammatory diseases, several NSAIDs and COX-2 selective inhibitors have been evaluated in terms of ROS scavenging activity in the past. For a comprehensive overview about assays for evaluating ROS scavenging activity the reader is referred to an excellent review by Magalhaes *et al.* [152]. Figure 4 shows NSAIDs and COXIBs in clinical use, which have been identified as antioxidant compounds.

The aryl propionic acid based NSAIDs naproxen, ketoprofen, flurbiprofen, ibuprofen, fenoprofen, indoprofen, and fenbufen were found to act as antioxidants in a variety of different ROS and RNS screening assays (O_2_^•−^, H_2_O_2_, ^•^OH, ^•^NO, ONOO^−^) as well as on a cellular level to scavenge human neutrophil derived ROS. Interestingly, under these experimental conditions only naproxen was able to scavenge ROO^•^ radicals [153]. A stimulation of neutrophil oxidative burst by NSAIDs has also been observed. While piroxicam and phenylbutazone were shown to decrease the effects of neutrophil O_2_^•−^ burst, indomethacin provoked a slight increase [154]. Phenylbutazone furthermore scavenges OCl^−^ at physiological concentrations of 50–100 µM [155]. The prodrug sulindac and its pharmacologically active metabolite sulindac sulfide are scavengers of O_2_^•−^, ^•^OH, ^•^NO, ONOO^−^; noteworthy, metabolism to the active form sulindac sulfide increases antioxidant potency and adds the capability to scavenge OCl^−^ [156].

Flufenamic acid, diclofenac, niflumic acid, tenoxicam, piroxicam, and indomethacin did not directly scavenge OCl^−^ but were found to prevent HOCl-derived chlorination by inhibition of myeloperoxidase [157]. Furthermore, the selective COX-2 inhibitors tenoxicam, lornoxicam, piroxicam, meloxicam, and the sulfonanilide nimesulide were found to scavenge effectively ^•^OH and OCl^−^ but not H_2_O_2_ [158]. Beside antioxidant capacity of nimesulide, also its main metabolite 4-hydroxynimesulide exerts ^•^OH, O_2_^•−^, and peroxide scavenging activities [159,160]. Higher ROO^•^ scavenging activity of acetaminophen compared to BHT was reported by Nam *et al.* [161]. The NSAID nimesulide was evaluated by Kullich *et al.* in an open pilot study on 20 patients suffering from osteoarthritis of the knee regarding its antioxidant capacity in humans, *i.e.*, on glutathione *S*-transferase-π (GST-π) levels [162]. During 3 week treatment with nimesulide 100 mg two times a day, GST-π levels significantly increased to a level comparable with the healthy control group giving evidence for its antioxidant efficacy *in vivo* in addition to its analgesic and anti-inflammatory action [162]. Tenoxicam was shown to exert protective effects on oxidative stress in Wistar albino rat brain, *i.e.*, decreasing the lipid peroxidation level and increasing GSH, GSH-Px, vitamin C and E (but not A and E) levels, as evaluated by intramuscular administration for 10 days at a dosage of 10 and 20 mg/kg bodyweight per day [163]. In a clinical setting, celecoxib and tenoxicam were evaluated in 25 patients with knee osteoarthritis which showed that both drugs did not change serum MDA levels or serum SOD and GSH-Px activity in patients but that celecoxib alone reduced nitrite and tenoxicam nitrite and XO [164].

Indomethacin has been extensively studied and revealed antioxidant activity against ^•^OH, O_2_^•−^, ^•^NO, ONOO^−^, and CCl_3_O_2_^•^. Furthermore, a lack of reactivity respectively a very low reactivity against OCl^−^ was determined and the ability to block ROS production from activated neutrophils [155,165,166]. Based on these findings, the indole containing NSAIDs indomethacin, acemetacin, and etodolac as well as the pyrrole-based derivatives tolmetin and ketorolac and the oxazole-based COX-1 inhibitor oxaprozin were compared in terms of their antioxidant capacity by Fernandes *et al.* [166]. All compounds exhibited scavenging activity against ROO^•^, ^•^OH, ^•^NO, and ONOO^−^ as well as a lack of reactivity against OCl^−^. Interestingly, the indole-based NSAIDs showed most potent scavenging of ^•^OH and, in comparison to the other test compounds exclusively, O_2_^•−^ scavenging pointing to the importance of delocalization of the lone electron pair in the indole moiety for their antioxidant capacity [166].

Celecoxib and to a higher extent nimesulide improved Gpx, SOD, and TAS (total antioxidant status) in hypercholesterolemic rabbits suggesting antioxidant potential of these inhibitors and that timely use would be beneficial to enhance the antioxidant defense system during hypercholesterolemia [167]. In contrast, renal damage in Wistar rats induced by celecoxib administration for 1 and 7 days, respectively, was accompanied by an increase in SOD, CAT, and GST and a decrease in GSH and reactive thiol levels suggesting that oxidative stress may play a role in this side effect [168]. In a study of ischemia/reperfusion induced injury in rat liver tissues, nimesulide was shown to effectively inhibit COX-2 activity and diminish oxidative stress as evident from increased GSH-levels and decreased levels of MDA, myeloperoxidase and DNA damage product 8-hydroxyguanine suggesting a benefit of nimesulide treatment in this pathology [169].

**Figure 5:**
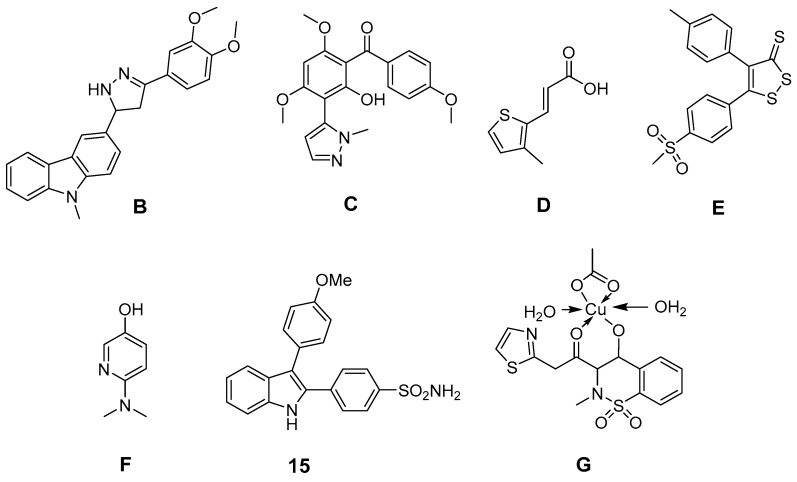
Novel synthetic COX-2 inhibitors showing antioxidant capacity in experimental models.

Further efforts were undertaken to design antioxidant COX-2 inhibitors (Figure 5). 3-Aryl-5-(9-methyl-3-carbazole)-1*H*-2-pyrazolines like **B**, synthesized by Bandgar *et al.*, showed, at 100 µM, COX-2 selective inhibition as well as 2,2-diphenyl-1-picrylhydrazyl (DPPH), O_2_^•−^ and ^•^OH scavenging activity [170]. The same group developed [2-hydroxy-4,6-dimethoxy-3-(2-methyl-2*H*-pyrazolyl)]-phenyl-methanone derivatives like **C** which showed unselective COX inhibition at 100 µM as well as DPPH, O_2_^•−^, and ^•^OH scavenging activity at the same concentration [171]. Pontiki *et al.* synthesized acrylic acid derivatives as inhibitors of lipoxygenase and COX-1 with antioxidant activity. (*E*)-3-(3-Methylthiophen-2-yl) acrylic acid (**D**) and its 5-methyl-substituted analog were found to be anti-inflammatory agents *in vivo* in a carrageenan-induced rat paw edema model [172]. These derivatives showed *in vitro* low (37%) respectively potent (75%) inhibition of COX-1 at a concentration of 100 µM as well as IC_50_ values to inhibit lipoxygenase in the high µM range. Both compounds showed high antioxidant activity for ^•^OH as well as for O_2_^•−^, but low to moderate activity in a DPPH, in an AAPH, in an heme dependent lipid peroxidation, and in an ABTS radical cation scavenging assay [172]. Diaryl-substituted isothiazoles, 3*H*-1,2-dithiole-3-thiones and 3*H*-1,2-dithiole-3-ones were developed as multiple target non-steroidal anti-inflammatory drugs (MTNSAIDs) by Scholz *et al.* [173]. As example 5-(4-(methylsulfonyl)phenyl)-4-*p*-tolyl-3*H*-1,2-dithiole-3-thione (**E**) was found to be an inhibitor with overall balanced inhibition profile of COX-1 (IC_50_ = 7 µM), COX-2 (IC_50_ = 9 µM), 5-LOX (IC_50_ approx. 10 µM), and ^•^OH (IC_50_ = 9 µM). The compound was furthermore identified *in vitro* and *in vivo* as inhibitor of the expression of macrophage adhesion complex-1 and adhesion and infiltration of leukocytes. Interestingly, the methylsulfonyl moiety was found to support ^•^OH scavenging activity in dithiothiones and dithiolones. Celecoxib was found to have an IC_50_ of approx. 500 µM to scavenge ^•^OH and thus to be a weak ^•^OH scavenger [173]. Pyridinol as well as pyrimidinol derivatives of acetaminophen were developed by Nam *et al.* and evaluated in terms of antioxidant activity in an AMVN and AIBN assay, COX-1 and LOX inhibition potency, and toxicity *in vitro* [161]. In comparison to acetaminophen, a decrease of reactivity as well as COX inhibition potency but increase of LOX inhibition potency was observed by replacement of a phenyl ring with one of these nitrogen containing heterocycles. Interestingly, the *N*,*N*-dimethylamino-substituted 3-pyridinol **F** was found to be a potent antioxidant and an inhibitor of COX-1 (IC_50_ 22 µM) for that, as discussed by the authors, a two-electron oxidation could lead to an alkylating agent that irreversibly inactivates the COX peroxidase active side [174]. In addition, indole-based COX-2 inhibitors have been designed and antioxidant activity of 2,3-diphenyl-1*H*-indole **15** has been described above. Other synthetic indoles will be described at the end of this section. Of note, also SOD mimicking compounds based on copper complexes of COX-inhibitors have been synthesized and evaluated. Cu(II)_2_ (3,5-diisopropylsalicylate)_4_ has been described to exert antioxidant activity and to act as radioprotector [99,100]. 4-Hydroxy-2-methyl-*N*-(2-thiazole)-2*H*-1,2-benzothiazine-3-carboxamide-1,1-dioxide (EX15) and its Cu(II) complex (**G**) were synthesized by Sherif *et al.* and evaluated in an adjuvant-induced rheumatoid arthritis model in rats [175]. The complex itself was not tested regarding its COX inhibition profile. However, both revealed antioxidant, analgesic and anti-rheumatoid effects. The ability of copper(II) ions to scavenge O_2_^•−^ was discussed by the authors as the most likely reason for the anti-inflammatory action of the complex [175].

**Figure 6:**
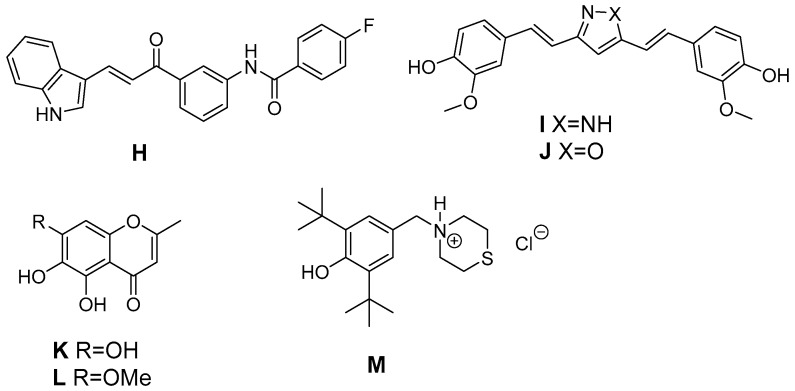
Antioxidant COX-2 inhibitors derived from natural leads.

In addition, natural antioxidants served as leads for the development of antioxidant COX-2 inhibitors (Figure 6). The antioxidant curcumin has a wide variety of positive effects on the inflammatory pathway, e.g., the suppression of NF-κB caused induction of COX-2 in Kupffer cells and lung cancer cells [176]. Asymmetrical indole curcumin analogs, *i.e.*, *N*-(3-(3-1*H*-indole-3-yl)acroyl)phenyl)-4-fluorobenzamide (**H**), were found to inhibit COX-2 (83% @ 10 µM) and to be moderate DPPH radical scavengers (44% @ 10 µM) and K_3_[Fe(CN)_6_] reducing agents (35% @ 10 µM) [177]. Pyrazole (**I**, X = NH) and isoxazole (**J**, X = O) based curcumin analogs have been synthesized by Selvam *et al.* to elucidate structure activity relationship regarding COX inhibition and antioxidant activity [178]. Both analogs were COX-1 selective inhibitors with radical scavenging activity in the DPPH assay like curcumin. However, the synthetic analogs showed a slightly improved COX-2 inhibitory activity (e.g., pyrazole: COX-1: 80% inhibition @ 100 µM, COX-2: 61% inhibition @ 100 µM). Naturally occurring chromones have been reported to exert anti-inflammatory, antioxidant, and a variety of other pharmacological activities targeting among others COX, NO production, and PKC. A series of stellatin derivatives (**K**, **L**) was synthesized by Gautam *et al.* to evaluate their COX inhibition profile as well as antioxidant capacity in a DDPH and an ABTS assay [179]. The novel stellatin derivatives showed only slight COX-2 selective inhibition having IC_50_ values in the µM range for COX-1 and COX-2. Interestingly, two compounds, both characterized by hydroxyl-groups at position 6 or 7 of the chromone, showed antioxidant capacity comparable to trolox and curcumin [179]. Ziakas *et al.* created a set of butylated hydroxytoluene derivatives. 2,6-Di-*tert*-butyl-4-thiomorpholin-4-ylmethylphenol hydrochloride (**M**), the most potent derivate of the series, was found to be a selective COX-1 inhibitor (IC_50_ COX-1 = 6.5 µM), an inhibitor of lipoxygenase and additionally an antioxidant as evaluated in a lipid peroxidation model and DPPH assay [180]. Of note, inhibition of COX-1 was observed only at 0.1 µM but not higher concentrations of arachidonic acid indicating the competitive nature of this inhibitor. The synthetic analog α-tocopheryl succinate (αTOS), showed a concentration dependent COX-2 inhibition (e.g., 63% at 30 µM), in contrast to tocopherol, as evaluated by measuring lipopolysaccharide (LPS) induced PGE_2_ formation in RAW264.7 cells [181].

**Figure 7:**
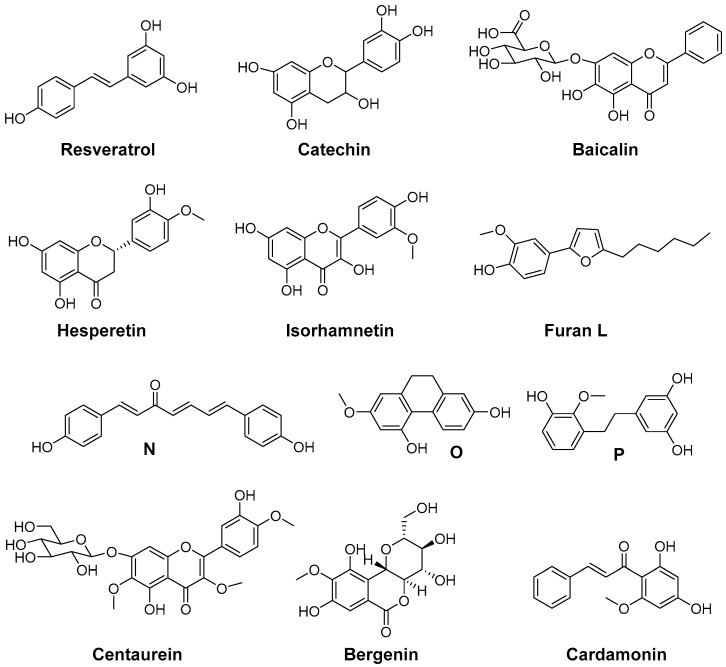
Antioxidant COX inhibitors isolated from plants.

Natural antioxidants (Figure 7) were found to have COX inhibitory activity as well, although the potency of these compounds is rather low. PGE_2_ and ROS production by microglial cells is associated with neuroinflammation. In this context, the natural antioxidant resveratrol, *trans*-3,5,4′-trihydroxystilbene, was investigated *in vitro* using microglial cells stimulated by lipopolysaccharide. At concentrations higher than 1 µM, resveratrol was able to inhibit the formation of ROS derived 8-*iso*-PGF_2α_ and COX-2 derived PGE_2_ formation thus targeting two important pathophysiological pathways [182]. Flavocoxid, a FDA regulated prescription in the United States, is an extract containing a mixture of catechin and baicalin. Among others, it reduces COX-2 expression as a consequence of NF-κB modulation as well as COX activity by inhibition of peroxidase activity and is additionally a LOX inhibitor and a strong antioxidant (oxygen radical absorbance capacity, ferric reducing/antioxidant power, peroxynitrite radical averting capacity). Open label clinical trials indicated efficacy of flavocoxid in patients with osteoarthritis together with a good overall and gastrointestinal tolerability [151,183]. The flavones hesperidin and its aglycon hesperetin were found to inhibit LPS-induced COX-2 gene expression at a concentration level of 250 µM and to exert ROS scavenging activity in an AIBN and benzoyl peroxide based assay [184]. Isorhamnetin, an antioxidant 3′-*O*-methylated metabolite of quercetin, was shown to exert its anti-inflammatory effects in part by reduction of COX-2 mRNA synthesis and protein expression [185]. Furan L was found to be a slightly selective and moderately potent COX-2 inhibitor having an IC_50_ in the µM range for COX-1 and COX-2, and to possess 5-LOX inhibition potency as well as low antioxidant activity compared to α-tocopherol. However, furan L showed cytoprotective effects on neuronal cells death induced by glutamate or lipopolysaccharide [186]. *Dioscorea opposite* (Dioscoreaceae) is widely used in China, Japan, and Korea as a traditional medicine with anti-inflammatory activity. Yang *et al.* determined the chemical constitution by isolating 19 aromatic compounds from the chloroform soluble fraction and tested the antioxidative and COX-2 inhibitory activity of these compounds [187]. Four selective and two unselective inhibitors of COX-2 with IC_50_ (COX-2) in the low mg/L level were identified which additionally exerted ROS scavenging activity in either both, DPPH and O_2_^•−^scavenging assay, or in one of them. The chemical structures of 3,3′,5-trihydroxy-2′-methoxy-bibenzyl (**N**), (4*E*,6*E*)-1,7-bis(4-hydroxyphenyl)-4,6-heptadien-3-one (**O**), and 9,10-dihydro-7-methoxy-2,5-phenanthrenediol (**P**) are shown as examples for a DPPH, a O_2_^•−^, and a DPPH and O_2_^•−^ scavenger, respectively, illustrating also the chemical variety of the isolated COX-2 selective compounds [187]. *Tridax procumbis L.* (Compositae) is used in traditional Indian medicine by application of the poultice of the whole plant because of its anti-inflammatory activity. Jachak *et al.* observed anti-inflammatory efficacy by all three selected extracts of the aerial parts of the plant in the carrageenan-induced rat paw edema assay *in vivo* and isolated three compounds as an attempt to identify COX inhibiting compounds [188]. In that course, the extract constituents centaurein and bergenin were found to be rather weak scavengers of ROS in the DPPH and 2,2'-azino-bis(3-ethylbenzothiazoline-6-sulphonic acid (ABTS) assay (IC_50_ > 100 µM), and to be weak inhibitors of COX-2 showing 42% and 21% inhibition at 100 µM, respectively, and a distinct selectivity for COX-1. The extracts were shown to exert unselective COX inhibition in the range of 38%–66% at a concentration of 50 µg/mL and ROS scavenging with IC_50_ in the range of 18–46 µg/mL which revealed that COX inhibition and ROS scavenging may be at least in part the mode of action of the anti-inflammatory extracts [188]. Cardamonin is a naturally occurring chalcone isolated from Zingiberous herbaceous plant species and traditionally used for its anti-inflammatory activity. It was found to be a scavenger of NO_2_^−^ and intracellularly produced ROS in LPS/interferon γ (IFN-γ) stimulated RAW264.7 cells, a suppressor of TNF-α synthesis and an inhibitor of COX-1 and COX-2 activity with IC_50_ in the low to middle µM level [174].

## 7. Indoles as Prominent Leads for the Development of Antioxidant Agents and Radioprotectors

The indole scaffold is part of a variety of molecules with biological importance like tryptophan, serotonin, ergot alkaloids, and plant hormones and regarded as privileged structure in medicinal chemistry [189,190,191]. Synthetic indole derivatives comprise compounds having, e.g., anti-cancer, antioxidant, anti-rheumatic, aldose reductase inhibitory, anti-bacterial, anti-fungal, anti-viral, anti-malarial, and anti-HIV activity [150]. Among this class, 2-arylindoles have been pointed out to be a promising sub-class with a manifold of biological activities [192]. Noteworthy, the most prominent indole compound due to its biological, antioxidant, and radioprotective activity is probably melatonin, which scavenges ^•^OH, ROO^•^, ONOO^−^ and protects against LDL oxidation [150,193,194,195]. The antioxidant capacity of indole-based compounds has extensively been investigated in structure activity relationship studies for melatonin and its derivatives [150,196,197,198] as well as other indole based compounds [144,150,194,199,200,201,202] in the past.

**Figure 8:**
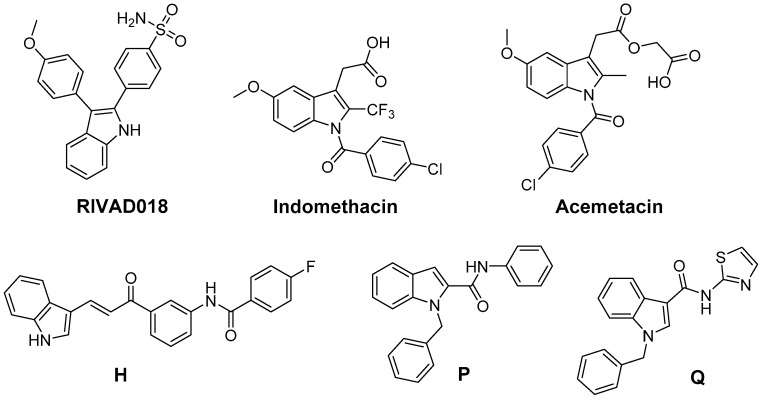
COX-2 inhibitors based on the indole scaffold with known antioxidant activity.

Beside 2,3-diphenyl-indole based COX-2 inhibitors like **RIVAD018**, the NSAIDs indomethacin and acemetacin as well as **H**, only some indole-based COX inhibitors have been evaluated regarding their antioxidative capacity (Figure 8). The ROS scavenging activity of a series of indole-2 and 3-carboxamides bearing a benzoyl, benzyl, or phenyl substituent at the indole nitrogen was evaluated by Ölgen *et al.* [203,204,205,206]. In these experiments, by varying, e.g., the amine component by a phenyl and a thiazolyl group, the ^•^OH scavenging phenyl derivative **P** and the O_2_^•−^, ^•^OH, and ^1^O_2_ scavenging thiazolyl-substituted compounds like **Q** were identified. A set of compounds was evaluated by the authors in terms of COX inhibition potency in a human whole blood assay which revealed COX-2 selective inhibition with IC_50_ in the low µM range of some *N*-phenyl- and *N*-thiazolyl-indole-3-carboxamides [206]. Analogously, *N*-substituted indole-2-carboxylic acid esters were developed by Ölgen *et al.* but these derivatives lacked COX-2 inhibition and showed weak COX-1 inhibition potency [207]. However, as later on evaluated by Kruk *et al.*, these compounds also exerted antioxidant capacity scavenging O_2_^•−^, ^•^OH, and ^1^O_2_ [208].

**Figure 9:**
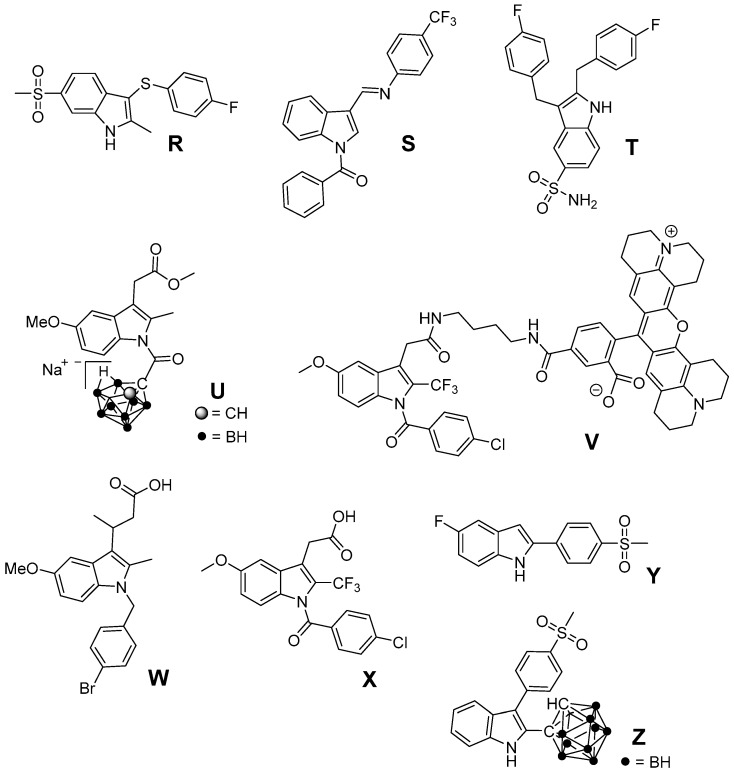
Indole-based COX-2 inhibitors which have not been evaluated regarding their antioxidant capacity.

Based on the finding that several structurally simple as well as complex indole-based compounds possess antioxidant potency due to inherent properties of the indole heterocycle, we suggest that also a variety of indole-based COX inhibitors, as discussed below, should display antioxidant properties (Figure 9). For instance, 3-arylthio-2-methyl-6-methylsulfonyl-1*H*-indole **R** as presented by Campbell *et al.* as well as *N*-benzoyl-substituted 3-[(phenylimino)methyl]indole **S** described by Kaur *et al.* were found to exhibit selective COX-2 inhibition in the nanomolar range [209,210]. The 5-aminosulfonyl-substituted 2,3-dibenzyl-1*H*-indole **T** was found to be a rather weak (67% inhibition of COX-2 at 50 µM) but selective COX-2 inhibitor [211]. Indomethacin represents a clinically used NSAID known to exert ROS scavenging activity [212,213]. Based on the fact that by esterification or amidation of indomethacin further COX-2 selective inhibitors can be obtained, several substituted indomethacin derivatives have been synthesized so far [214,215,216,217,218,219]. Recently, the novel COX-2 selective and, noteworthy, hydrophilic indomethacin ester derivative **U** was identified by replacement of the benzoyl group with a for COX-2 inhibitors novel pharmacophore, a nido-carbaboran cluster [220]. Of note, the indomethacin-ROX conjugate fluorocoxib-A as well as its trifluoromethyl-analog **V** represent promising COX-2 selective inhibitors having fluorescent properties suitable for fluorescence-based imaging of COX-2 *in vivo* [219,221]. Further COX-2 selective indomethacin analogs with a free carboxylic acid group have been synthesized, e.g., by extension of the acyl side chain (**W**) [222] or by replacement of the methyl with an trifluoromethyl group (**X**) [223]. A selective COX-2 inhibition by 5-fluoro-2-(4-methylsulfonylphenyl)-1*H*-indole (**Y**, IC_50_ COX-2 = 0.1 µM; IC_50_ COX-1 = 12.5 µM) was described by Zarghi *et al.* [224]. Of note, Suzen *et al.* described DPPH and O_2_^•−^ scavenging as well as inhibition of lipid peroxidation by structurally similar 2-phenylindole derivatives [144]. Beside antioxidant 2,3-diphenyl-based COX-2 inhibitors like **RIVAD018** that have been discussed above, we synthesized 2-carbaboranyl-3-phenyl-1*H*-indole derivatives and found abolished COX-2 inhibition by replacement of the 2-phenyl ring with this pharmacophore. However, the *ortho*-carbaboranyl-substituted derivative **Z** of this class was found to be a COX-1 inhibitor (IC_50_ COX-1 = 1.6 µM; IC_50_ COX-2 > 4 µM) [225]. Although these indole-based COX inhibitors have not been identified as antioxidants yet, the antioxidant capacity of other indole-based compounds suggests potent ROS scavenging for these compounds as well so that further studies are warranted.

Finally, it should be pointed out that within all antioxidant COX-2 inhibitors presented in the two previous chapters some compounds were described as radiosensitizing or radioprotecting agent as well, but this property has not been considered for their evaluation in RT-based settings. Hence, the impact of ROS scavenging on the radioprotective effect during combined treatment with these inhibitors and RT was not determined and still remains unclear. In this regard, indomethacin and meloxicam are compounds that have been described as radiosensitizers and radioprotectors. Nimesulide was found to increase radiosensitivity and flurbiprofen was described as radioprotector. Results showing that SOD-mimicking Cu(II)_2_ (3,5-diisopropylsalicylate)_4_ exerted radioprotection *in vivo* and that the ROS scavenging and COX-2-selective 2,3-diphenyl-indole **RIVAD018** showed radioprotective effects *in vitro* hold promise that antioxidant COX-2 inhibitors can act as double-edged swords in RT.

## 8. Concluding Remarks

This review focused on recent efforts in development and characterization of antioxidant COX-2 inhibitors intended or deemed appropriate to be used for attenuation of radiation therapy-associated damage of normal tissue. However, although both the role of reactive oxygen/nitrogen species and inflammation-associated processes as a pathophysiological entity in RT is widely accepted, a thorough investigation of the potential of antioxidant COX inhibitors, preferentially targeting COX-2, in the treatment of acute and late adverse effects of radiation therapy has not been undertaken. In order to stimulate and strengthen in-depth discussion on this exciting topic in a hypothesis-driven approach several preclinical and clinical data on COX-2 inhibitors, which already have been tested regarding their radioprotective potential, were discussed.

The radioprotective action, particularly, was ascertained to the inhibition of the prostaglandin synthesis and, directly or indirectly linked with the ability of NSAIDs to arrest cells in the G0 or G1 phase where they are less sensitive to radiation damage [92], and/or by stimulation of the hematopoietic recovery [89]. Additionally, several COX-2 inhibitors with antioxidant properties have been described. The combination of these strategies revealed first promising results *in vitro* and *in vivo*. It has to be noted that COX-1 and COX-2 inhibitory potency as well as specific antioxidant properties are not the only parameters that are important for radioprotection. NSAIDs appear to have tissue specific responses [90] as it can be anticipated for COXIBs as well. Hence, the pharmacology of the drug, more specifically its biodistribution and metabolism, has to be taken into account. For example, 2,3-diphenyl-1*H*-indole **RIVAD018** is secreted hepatobiliary [139,141] and would unlikely be able to protect radiation induced renal damage. In addition, differences in pathophysiological processes after single *vs.* fractionated doses, low *vs.* high doses as well as the response of different tissues like skin or mucosa have to be taken into account [129]. Furthermore, we feel that research would highly benefit from the determination of the most important parameters for antioxidant and COX activity, and the standardization of the used *in vitro* and *in vivo* assays. Detailed knowledge of antioxidant activity of COX-2 inhibitors also could promote synergistic pharmaceutical combinations. In this regard, intuitively, combination of ACE inhibitors and antioxidant COX-2 inhibitors should be radioprotective, e.g., by preventing radiation fibrosis the in cardiopulmonary system [226,227].

This “double-edged sword” conceptional idea may be extended along two axes. Clearly, novel radioprotective agents should be developed in a way that they specifically target normal cells without conferring protection to tumor cells. In this regard, antioxidant COX-2 inhibitors still could be considered as tumor radiosensitizing agents because of their potent inhibition of COX-2. Furthermore, also the modulation of a variety of COX-2 independent pathways by COX inhibitors has been described as a reason for radiosensitization of different tumor cell lines. Under conditions of high dose ROS/RNS generation in tumor tissue this enzyme-targeted action possibly prevails the antioxidative action. Thus, it is conceivable to use antioxidant COX-2 inhibitors as radioprotectants of normal tissue and radiosensitizers of cancer tissue in parallel. In a broader sense, another COX-2 targeting approach using nitric-oxide (NO^•^)-releasing COX-2 inhibitors could contribute in terms of a new class of bifunctional radioprotecting and radiosensitizing agents. Here, the release of an additional radical, NO^•^, is likely to enhance intended radiation damage of tumor cells. On the other hand, NO^•^ as a key factor for maintenance of, e.g., endothelial homeostasis, might counteract the radiation-induced endothelial dysfunction [228,229].

Antioxidant COX-2 inhibitors have the potential to attenuate radiation-induced late effects. However, no compound has been thoroughly investigated starting from *in vitro* leading to controlled *in vivo* settings so far. In this regard, this review raised the question, and attempted to find answers, whether antioxidant COX-2 inhibitors might be a useful double-edged sword to support RT of cancer patients. Although the current data situation supports this hypothesis in principle, an answer cannot be conclusively given and the proof will be a continuous and interesting challenge of further preclinical and clinical studies.
